# Significance testing in ridge regression for genetic data

**DOI:** 10.1186/1471-2105-12-372

**Published:** 2011-09-19

**Authors:** Erika Cule, Paolo Vineis, Maria De Iorio

**Affiliations:** 1 Department of Epidemiology and Biostatistics, School of Public Health, Imperial College London, London, UK

## Abstract

**Background:**

Technological developments have increased the feasibility of large scale genetic association studies. Densely typed genetic markers are obtained using SNP arrays, next-generation sequencing technologies and imputation. However, SNPs typed using these methods can be highly correlated due to linkage disequilibrium among them, and standard multiple regression techniques fail with these data sets due to their high dimensionality and correlation structure. There has been increasing interest in using penalised regression in the analysis of high dimensional data. Ridge regression is one such penalised regression technique which does not perform variable selection, instead estimating a regression coefficient for each predictor variable. It is therefore desirable to obtain an estimate of the significance of each ridge regression coefficient.

**Results:**

We develop and evaluate a test of significance for ridge regression coefficients. Using simulation studies, we demonstrate that the performance of the test is comparable to that of a permutation test, with the advantage of a much-reduced computational cost. We introduce the *p*-value trace, a plot of the negative logarithm of the *p*-values of ridge regression coefficients with increasing shrinkage parameter, which enables the visualisation of the change in *p*-value of the regression coefficients with increasing penalisation. We apply the proposed method to a lung cancer case-control data set from EPIC, the European Prospective Investigation into Cancer and Nutrition.

**Conclusions:**

The proposed test is a useful alternative to a permutation test for the estimation of the significance of ridge regression coefficients, at a much-reduced computational cost. The *p*-value trace is an informative graphical tool for evaluating the results of a test of significance of ridge regression coefficients as the shrinkage parameter increases, and the proposed test makes its production computationally feasible.

## Background

Genetic data collected in case-control or cohort studies of well-defined disease phenotypes can be used to identify genetic variants, typically single nucleotide polymorphisms (SNPs), associated with disease status. In a genetic association study, the data consist of genotypes and corresponding phenotypes from large numbers of individuals with the disease phenotype of interest (cases) and disease-free controls. A significant difference in the frequency of a genetic variant between the case and control groups is taken to be indicative of an association between that variant (or, more probably, a nearby causal variant correlated with the typed variant) and the phenotype of interest. A framework also exists for analysing a continuous phenotypes. Once one or more associated variant have been identified, they can be included in predictive models for the estimation of disease risk in individuals for whom the (potentially future) disease status is unknown. Technological developments, including commercially available chips for typing millions of genetic variants simultaneously, and next-generation sequencing technologies such as those used in the 1000 Genomes Project [1], are enabling the collection of large amounts of genetic data from large numbers of individuals which means that analysis of contemporary genetic data sets involves the study of high-dimensional data.

A number of statistical approaches have successfully been used to investigate the strength of association between genetic variants and a phenotype of interest. These methods include testing the significance of the association of each variant with the phenotype independently using standard univariate tests (such as the Cochran-Armitage test for trend [2] or Fisher's exact test). However, there are disadvantages to relying on univariate methods for the analysis of genetic data. The data from a genome-wide association study typically consists of thousands or millions of SNPs, and this large number of predictors introduces a multiple testing problem. Univariate methods therefore require stringent significance thresholds due to the large number of tests being undertaken to prevent a high false-positive rate [3]. Further, univariate methods fail to take into account the combined effect of multiple SNPs. It is plausible that many genetic variants together contribute to the phenotype being studied [4], and one advantage of using multivariate methods is that they allow for the study of the combined effect of multiple SNPs. Multivariate methods allow for the control of confounding variables, as in the lung cancer replication study in this paper, where gender, smoking status and age were included as unpenalised covariates in the model.

Using multivariate methods, the regression model can be extended to include, for example, interaction or higher order terms, and in such a case a penalised regression approach would be appropriate [5]. Penalised regression methods have been applied to genetic data [6,7]. Among a number of regression approaches used for prediction in high-dimensional data, ridge regression has been shown to perform best in terms of prediction error [8]. Ridge regression has successfully been used to analyse genetic data where SNPs were in high LD [9]; it is the test of significance used by Malo, Libiger & Schork that we evaluate here.

To begin, we consider two regression models commonly used in the analysis of genetic data - the linear and the logistic regression models, as follows.

The standard linear regression model is given by

(1) Y=Xβ+ε

where **Y **is a (*n *× 1) vector of dependent variables, *Y_i_*, *i *= (1, ..., *n*) and **X **is a (*n *× *m*) matrix of predictors. ***β ***is a (*m *× 1) vector of regression coefficients *β_j_*, *j *= (1, ..., *m*) and ***ε ***is a (*n *× 1) vector of normally distributed random errors, with $\varepsilon_{i}\overset{\text{iid}}{\sim }N\left(0,\sigma^{2}\right)$. An example would be a model of the relationship between a continuous phenotype (such as blood pressure or plasma lipid concentration) measured in *n *individuals, and the genotype of these *n *individuals at *m *SNPs.

The ordinary least squares estimator for ***β ***is given by

(2) $$\hat{\beta }={\left(X^{\prime }X\right)}^{-1}X^{\prime }Y$$

The significance of individual OLS regression coefficients ${\hat{\beta }}_{j}$ in a multiple regression model can be estimated using a Wald test. The test statistic is

$$T_{0}=\frac{{\hat{\beta }}_{j}}{se\left({\hat{\beta }}_{j}\right)}$$

where $se\left({\hat{\beta }}_{j}\right)$ is an estimate of the standard error of the *j*^th ^regression coefficient. Under the null hypothesis $H_{0}:{\hat{\beta }}_{j}=0$, *T*_0 _follows a Student *t *distribution with *n *- *m *degrees of freedom.

Binary outcomes commonly arise in biomedical data where they may represent, for example, cases and controls. In the logistic regression model, **Y **is an *n*-dimensional vector of response variables taking values 0 (controls) or 1 (cases), and **X **the *n *× *m *matrix of explanatory variables, as before. For the *i*^th ^individual we denote

$$x_{i}=\left(X_{i1},\ldots ,X_{im}\right)$$

The *i*^th ^response *Y_i _*is a Bernoulli variable with probability of success equal to *p_i_*. The logistic regression model relates the probability *p_i _*that the *i*^th ^observation is a case to the predictor variables as

(3) $$P\left(Y_{i}=1|x_{i}\right)=p_{i}=\frac{e^{x_{i}\beta }}{1+e^{x_{i}\beta }}$$

where ***β ***is a vector of parameters to be estimated.

The significance of individual logistic regression coefficients, ${\hat{\beta }}_{j}$, can be estimated using the test statistic

$$T_{0}=\frac{{\hat{\beta }}_{j}}{se\left({\hat{\beta }}_{j}\right)}$$

where $se\left({\hat{\beta }}_{j}\right)$ is an estimate of the standard error of the *j*^th ^regression coefficient. Under the null hypothesis that *β_j _*= 0, *T*_0 _asymptotically follows a standard normal distribution.

Genetic data often comprises more predictor variables, *m*, than observations, *n*. In such a situation, unique maximum likelihood estimates of regression parameters do not exist. Further, collinearity in the predictors, due to linkage disequilibrium (LD) in genetic data, which typically increases with the increasing density of available markers, results in unstable maximum likelihood estimates of regression coefficients.

An extensive literature exists on the application of modified regression techniques to the analysis of high-dimensional data. Penalised regression constrains the magnitude of the estimated regression coefficients, allowing their estimation when ordinary least squares (OLS) estimates cannot be obtained. In a Bayesian context, these techniques are equivalent to the specification of a particular prior distribution on the coefficients. For example, Lasso regression [10] constrains the sum of the absolute value of the regression coefficients to be less than a constant. This is equivalent to imposing a double exponential prior centred at zero on the coefficients. Lasso regression can estimate some coefficients to be exactly zero, permitting dimension reduction in the model. Hoggart, Whittaker, De Iorio & Balding [6] considered a modified Lasso regression approach for the identification of causal SNPs in genome-wide or resequenced data, with the aim of identifying regions of association whilst considering all SNPs simultaneously. When SNPs are in high LD, their method offers improvement over both single-SNP analysis and Lasso regression in terms of the power to detect causal variants, and a notable improvement over single-SNP analysis in terms of false-positive rate.

Ridge regression [11] is a another penalised regression approach, in which a penalty is applied to the sum of the squared parameter estimates. Ridge regression has been used in a number of large-scale data analysis scenarios, including marker-assisted selection [12], expression data analysis [13], and genetic association studies when SNPs are in high LD [14]. From a Bayesian viewpoint, ridge regression can be considered as standard multiple regression with the coefficients estimates having a prior distribution that is normal with mean zero and known variance [15]. In genetic epidemiology, it is desirable to estimate the strength of the association between a variant and a phenotype. This is problematic when using ridge regression which, unlike other penalised regression approaches, does not reduce the number of parameters in the model, nor estimate the significance of each fitted coefficient.

A test of significance for coefficients estimated using ridge regression, based on an approximation of their distribution under the null hypothesis, was proposed by Halawa & El Bassiouni [16]. The test was originally developed and evaluated for data with continuous outcomes, when different methods were used to compute the shrinkage parameter, *λ*, which controls the degree of shrinkage of the regression coefficients and hence their distribution under the null hypothesis. Malo, Libiger & Schork [9] used the same test in an evaluation of the applicability of ridge regression as a means of accommodating LD in association studies. They used the test in a comparison of the performance of ridge regression, multiple regression and single-SNP analysis when SNPs are in varying degrees of LD. They found that ridge regression identified different SNPs as associated with phenotype compared to single-SNP analysis or multiple regression. However, they did not consider the performance of the test itself.

In this paper, we evaluate the performance of a test of significance for ridge regression coefficients. Our test is based on the test proposed by Halawa & El Bassiouni [16]. We extend the test, making it applicable in the *m > n *scenario that is common in contemporary genetic data sets. We evaluate the performance of the test in simulation studies, using scenarios representative of realistic high-desnity genetic marker data, considering a range of data set dimensions and degrees of shrinkage.

Ridge regression has also been applied in the logistic regression framework [17]. We extend the test proposed by Halawa & El Bassiouni [16] to the logistic ridge regression model, and again evaluate the test in a range of simulation scenarios at different values of *λ*.

In both linear and logistic ridge regression, we compare the approximate test of significance to a permutation test. We view the permutation test as a benchmark as it produces an estimate of the null distribution of the parameter estimates. However, the permutation test is computationally intensive and becomes more so when data are high-dimensional. The test we propose makes it feasible to estimate significance with a much lower computational burden.

We introduce the *p*-value trace, a plot of the negative logarithm of the *p*-values of the ridge regression coefficients with increasing shrinkage parameter. This plot enables the visualisation of the relative change in significance of each coefficient, and facilitates the identification of predictors most affected by increased penalisation in terms of significance.

We apply the approximate test of significance for logistic ridge regression coefficients to a lung cancer data set, demonstrating the utility of the test when correlation exists among the predictors.

This paper is organised as follows. We first describe the approximate test of significance and the permutation test to which it is compared. We then describe the simulation studies used in this paper. In the Results section we evaluate the performance of the proposed test in a range of simulation scenarios. Further, we apply the test to a lung cancer case control data set. In the Discussion we discuss the results and potential applications for the test.

## Methods

### Significance testing in linear ridge regression

Ridge regression replaces the OLS estimator $\hat{\beta }$ (equation (2)) with the ridge regression estimator [11]:

(4) $${\hat{\beta }}^{\lambda }={\left(X^{\prime }X+\lambda I\right)}^{-1}X^{\prime }Y$$

Where collinearity exists in **X**, the OLS estimates of ***β ***can be unstable, having large variance. Hoerl & Kennard [11] demonstrate that there exists a value of *λ *for which the ridge regression estimates ${\hat{\beta }}^{\lambda }$ have smaller mean square error (MSE) than the OLS estimates. Where *m > n*, OLS estimates of ***β ***cannot be obtained because the matrix **X**'**X **is singular. The addition of the constant *λ *to the diagonal of the **X***'***X **matrix makes it invertible, so ridge regression estimates can be obtained.

Halawa & El Bassiouni [16] use simulation studies to investigate applications of a 'non-exact' *t*-type test for the individual coefficients of a linear regression model fitted using ridge regression, based on the *t*-test above. The test statistic is

$$T_{\lambda }=\frac{{\hat{\beta }}_{j}^{\lambda }}{se\left({\hat{\beta }}_{j}^{\lambda }\right)}$$

where ${\hat{\beta }}_{j}^{\lambda }$ is the estimate of the *j*^th ^regression coefficient under the ridge regression model, and *se*(${\hat{\beta }}_{j}^{\lambda }$) is an estimate of the standard error.

Estimates of the standard error of the *j*^th ^element of ${\hat{\beta }}^{\lambda }$ are obtained as the square root of the *j*^th ^element of the diagonal of the covariance matrix

$$\text{Var}\;\left({\hat{\beta }}^{\lambda }\right)=\sigma^{2}{\left(X^{\prime }X+\lambda I\right)}^{-1}X^{\prime }X{\left(X^{\prime }X+\lambda I\right)}^{-1}$$

In practise, *σ*^2 ^is replaced by its estimate, given by the residual mean square of the ridge model:

(5) $${\hat{\sigma }}^{2}=\frac{{\left(Y-X\hat{\beta }\right)}^{\prime }\left(Y-X\hat{\beta }\right)}{\nu }$$

*ν *is the residual effective degrees of freedom. Halawa & El Bassiouni [16] use *ν *= *n *- *m*. However, when *m > n *this gives a negative estimate of the residual mean square. Instead, we use the definition of residual effective degrees of freedom given in Hastie & Tibshirani [18], which makes use of the "hat matrix", **H**:

(6) $$\hat{Y}=X{\left(X^{\prime }X+\lambda I\right)}^{-1}X^{\prime }Y$$

(7) =HY

**H **is termed the 'hat matrix', because it 'puts the hat on' **Y**, transforming it to **Ŷ**. Degrees of freedom for error are defined as

(8) $$\nu =n-\text{tr(}2H-HH^{\prime })$$

In linear regression, the hat matrix reduces to **H **= **X **(**X**'**X**)^-1 ^**X**' and *n *- tr (2**H **- **HH**') reduces to *n *- *m*. The test statistic *T_λ _*is assumed to follow a Student *t *distribution as in standard multiple linear regression. However, the effective number of parameters of the penalised regression fit is smaller than *m*. Hastie and Tibshirani define tr (**H**) as the degrees of freedom taken up by the penalised regression fit [18].tr (**H**) reduces to *n *- *m *in ordinary linear regression. Then, *T_λ _*is assumed to follow a Student *t *distribution with *n *- *tr *(**H**) degrees of freedom.

In the case of large sample size, as is typically the case in genetic data, the distribution of the test statistic is asymptotically normal, as noted by Halawa & El Bassiouni [16]. We compared the significance levels of the approximate test assuming both a normal and a Student *t*-distribution of the test statistic and found that the results were substantially identical. Therefore we assume that under *H*_0_, $T_{\lambda }\sim N\left(0,1\right)$ and use the normal distribution to test the significance of ridge regression coefficients. The results from the corresponding tests assuming that under *H*_0_, *T_λ _*~ *t_n _*_- tr(_**_H_**) are provided in an Additional File. See Additional File 1, Tables S1 and S2.

### Significance testing in ridge logistic regression

Ridge regression has been applied to the logistic regression model [17,19]. Cessie & van Houwelingen [17] show how ridge regression can be used to improve the parameter estimates in logistic regression when the number of predictors is relatively large or highly correlated. They discuss different ways of choosing the shrinkage parameter to minimize prediction error. Vago & Kennedy [19] apply ridge logistic regression to a clinical data set.

In logistic ridge regression, the log-likelihood function is penalised with the penalty applied to the *L*_2 _norm of ***β ***[19]. Maximum likelihood estimates of ***β ***are obtained by maximising the logarithm of the likelihood function [19], typically using the Newton-Raphson algorithm. The approximate test statistic is

$$T_{\lambda }=\frac{{\hat{\beta }}_{j}^{\lambda }}{se\left({\hat{\beta }}_{j}^{\lambda }\right)}$$

Standard errors of the coefficient estimates are obtained as the square roots of the *j*^th ^element of the diagonal of the covariance matrix. This matrix is estimated from the final Newton-Raphson iteration:

$$\begin{array}{lll} \text{Var}\left({\hat{\beta }}^{\lambda }\right) & =\text{Var}\left[{\left(X^{\prime }WX+2\lambda I\right)}^{-1}X^{\prime }Wz\right]\; & \text{(1)}\\ & ={\left(\frac{\partial^{2}\ell }{\partial \beta \partial \beta^{\prime }}\right)}^{-1}I\left(\beta \right){\left(\frac{\partial^{2}\ell }{\partial \beta \partial \beta^{\prime }}\right)}^{-1}\; & \text{(2)}\\ & ={\left(X^{\prime }WX+2\lambda I\right)}^{-1}\left(X^{\prime }WX\right){\left(X^{\prime }WX+2\lambda I\right)}^{-1}\; & \text{(3)}\\ & \; & \text{(4)} \end{array}$$

where *I *(***β***) is the observed information matrix, ℓ is the (penalised) log-likelihood, and **W **is the weight matrix:

$$\begin{array}{llll} W & =\text{diag}\left[\hat{p_{i}}\left(1-\hat{p_{i}}\right)\right]\; & & \;\\ \hat{p_{i}} & =\frac{e^{x_{i}{\hat{\beta }}^{\lambda }}}{1+e^{x_{i}{\hat{\beta }}^{\lambda }}}\; & & \; \end{array}$$

**z **is an *n *× 1 column vector with elements

$$z_{i}=\text{logit}\left[{\hat{p}}_{i}\right]+\frac{Y_{i}-{\hat{p}}_{i}}{\hat{p}\left(1-{\hat{p}}_{i}\right)}$$

Again we assume that under *H*_0_, *$T_{\lambda }\sim N\left(0,1\right)$*and use the normal distribution to test the significance of ridge regression coefficients.

### Permutation test

To evaluate the proposed test, we compare its performance to that of a permutation test, which we view as a benchmark. In the permutation test, to obtain a null distribution of the regression coefficients, the elements of the outcome vector **Y **are randomly permuted. The ridge regression model is fitted using the permuted observations, to obtain ridge regression coefficients. By performing 1000 such permutations, a null distribution of the regression coefficients is generated. The permutation test *p*-value is calculated as the proportion of regression coefficients from the null distribution greater than or equal in absolute value to the absolute value of the coefficient fitted to the true (non-permuted) data.

### Choice of shrinkage parameter

Hoerl & Kennard [11] present an existence theorem for ridge regression. They demonstrate the existence of a value of the shrinkage parameter *λ *in equation (4) which will give estimates ${\hat{\beta }}^{\lambda }$ with smaller mean squared error than the OLS estimates ***$\hat{\beta }$***given in (2).

However, to date no analytical method to find the 'best' value of *λ *in terms of minimising MSE has been determined. A number of data-driven methods have been proposed. These methods aim to determine a value of *λ *based on the data that will result in estimates of ***${\hat{\beta }}^{\lambda }$***with improved mean squared error properties. For example, Hoerl, Kennard & Baldwin [20] propose the following as an estimate of *λ*, with ${\hat{\sigma }}^{2}$ and ***$\hat{\beta }$***taken from the OLS estimates:

$$\lambda_{\text{HKB}}=\frac{m{\hat{\sigma }}^{2}}{{\hat{\beta }}^{\prime }\hat{\beta }}$$

An alternative estimate was suggested by Lawless & Wang [21], based on the adoption of the Bayesian perspective mentioned above. Cross-validation based methods have also been proposed in the literature [22].

**Figure 1:**
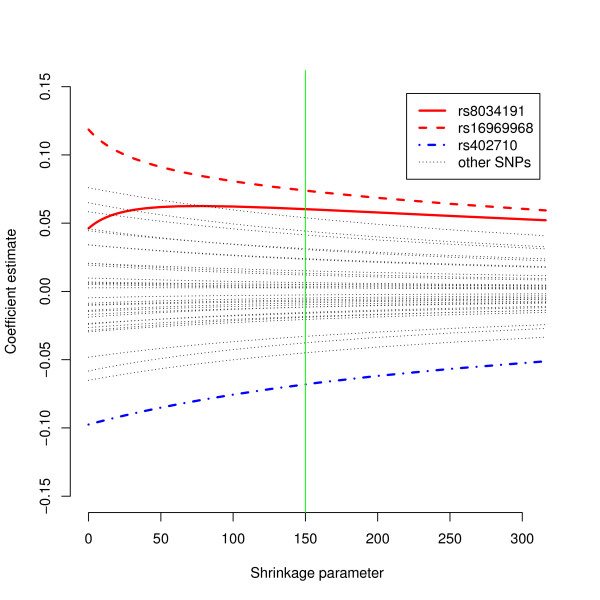
**Ridge trace - Lung cancer data**. Change in ridge regression coefficients with increasing shrinkage parameter. The three SNPs that have previously been shown to be associated with lung cancer risk, rs8034191, rs16969968 and rs402710, are shown in bold. Other SNPs are represented by dotted lines. The vertical line is at *λ *= 150, where the ridge estimates stabilise.

Hoerl & Kennard [11] introduce the ridge trace, a plot of the estimates ***${\hat{\beta }}^{\lambda }$***as *λ *increases from zero - see for example Figure 1. They propose choosing *λ *corresponding to the region on the ridge trace at which estimates of ${\hat{\beta }}^{\lambda }$ no longer change significantly as *λ *increases further.

**Figure 2:**
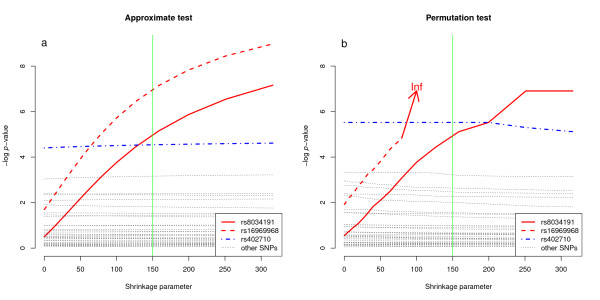
*****p***-value traces - Lung cancer data**. Change in *p*-values of ridge regression coefficients in lung cancer data with increasing shrinkage parameter. *p*-values are plotted on the *- *log scale. The three SNPs that have previously been shown to be associated with lung cancer risk, rs8034191, rs16969968 and rs402710, are shown in bold. Other SNPs are represented by dotted lines. The vertical line is at *λ *= 150, where the ridge estimates stabilise. **a **approximate *p*-values, **b **permutation test *p*-values.

Following the ridge trace of Hoerl & Kennard [11], we introduce a plot of *p*-values of the regression coefficients against *λ *as *λ *increases from zero (Figure 2). We refer to this plot as a '*p*-value trace'. This *p*-value trace enables the visualisation of the change in *p*-values of the regression coefficients with increasing shrinkage.

### Simulation study

The proposed test was evaluated using simulated genotype data. FREGENE software [23,24] was used to simulate a population of haplotypes. FREGENE simulates the forwards-in-time evolution of sequence-like genetic data. The forward-in-time simulation allows demographic and selection scenarios to be implemented and recombination to be modelled. Haplotypes used in this study are taken from a simulation representing the neutral evolution of 10,500 individuals over 200,000 generations, with recombination parameters that realistically model recombination in humans. This results in a population of 21,000 haplotypes. The simulated haplotypes, together with details of the simulation, are available to download from http://www.ebi.ac.uk/projects/BARGEN/[25].

Based on these haplotypes, genotypes and corresponding phenotypes were generated as follows. A range of scenarios were considered, comprising *n *individuals at *m *SNPs.

1. Designate one SNP as the causal SNP, selected at random from those with population minor allele frequency in the range 0.10 - 0.15. A subregion of *m *SNPs from the 1 Mb region, containing this causal SNP, is used for the genotype simulation. The subregion is selected at random to be of length *m *and to contain the designated causal SNP. Thus the correlation structure among the subregion depends on the correlation structure of the haplotype region at that point. In the resultant sample, correlation with the causal SNP ranges from low to perfect (*r*^2 ^= 1).

2. Sample two haplotypes (with replacement) from the population of 21,000 haplotypes. Sum the minor allele count at each SNP to form a genotype.

3. Simulate the phenotype for this individual.

Continuous phenotypes were generated as $Y_{i}\sim N\left(\mu ,\sigma^{2}\right)$.

Case-control phenotypes were generated following the liability model used by [26]. The penetrance function, *f_k_*, is the probability of being a case, Pr (*Y_i _*= 1) given having *k *copies of the minor allele at the causal SNP. The genotype relative risk, *r*, is *f*_1 _/*f*_0 _and *K *is the population prevalence. Then, with the population frequency of the minor allele of the causal SNP as *p*, under an additive genetic model, *f*_0 _= *K/*(1 - 2*p *+ 2*pr*), *f*_1 _= *rf*_0 _and *f*_2 _= 2*r f*_0 _- *f*_0_. A sample of *n/*2 cases and *n/*2 controls was generated by generating an individual genotype as described above, then assigning the individual to be a case with probability *f_k _*and a control otherwise. This process is repeated until *n/*2 cases and *n/*2 controls are obtained.

4. Record the minor allele count (0, 1, 2) at the *m *SNPs for the *i*^th ^individual, giving rise to an *n *× *m *matrix of minor allele counts.

Ridge regression coefficients were fitted to data with continuous outcomes using lm.ridge from the package MASS in R [27] for both the simulated data and the permutation test.

Estimates of regression coefficients under logistic ridge regression models were computed using the Newton-Raphson algorithm.

In the case of both continuous and binary outcomes, SNPs that were invariant in the sampled genotypes were removed from the data, and genotypes were standardised, prior to analysis.

The two tests were evaluated using the true positive rate (TPR) and false positive rate (FPR), averaged over all the replicates for each simulation scenario. We define TPR to be the proportion of causal SNPs, as designated in the data simulation, significantly associated with phenotype at the nominal threshold *α *= 0.05. TPR is not reported for the null simulations, as there is no causal SNP associated with phenotype in these data. We define FPR to be the proportion of non-causal SNPs significantly associated with phenotype at the same significance threshold.

## Results and Discussion

### Null Simulation

Genotypes and corresponding phenotypes were generated in two different sized data sets: (1) *n *= 500, *m *= 20 and (2) *n *= 1000, *m *= 1000. In generating the null data, no SNP was designated the causal SNP. Continuous phenotypes were generated as $Y_{i}\sim N\left(0,1\right)$; binary phenotypes were generated as *Y_i _*~ Binom(1, 0.5). False positive rates are reported at the nominal significance threshold *α *= 0.05. Four values of the ridge parameter *λ *were used.

Results of the null simulations are shown in Table 1. We find that approximate test gives similar results to the permutation test in terms of false positive rate, especially in the case of continuous outcomes.

**Table 1:** Performance comparison in null simulation

			Shrinkage Parameter							
			Approximate test				Permutation test			
			**0.1**	**1**	**10**	**100**	**0.1**	**1**	**10**	**100**
**Individuals**	**SNPs**	**Outcome**								
500	20	Continuous	0.066	0.066	0.066	0.075	0.066	0.066	0.066	0.075
		Binary	0.021	0.021	0.041	0.067	0.027	0.027	0.033	0.052
1000	1000	Continuous	0.050	0.049	0.050	0.046	0.051	0.052	0.050	0.046
		Binary	0.118	0.092	0.066	0.053	0.054	0.056	0.053	0.051

False positive rates at the nominal significance threshold *α *= 0.05 in null datasets. In each scenario, results are averaged over ten replicates

### Continuous Phenotypes

**Figure 3:**
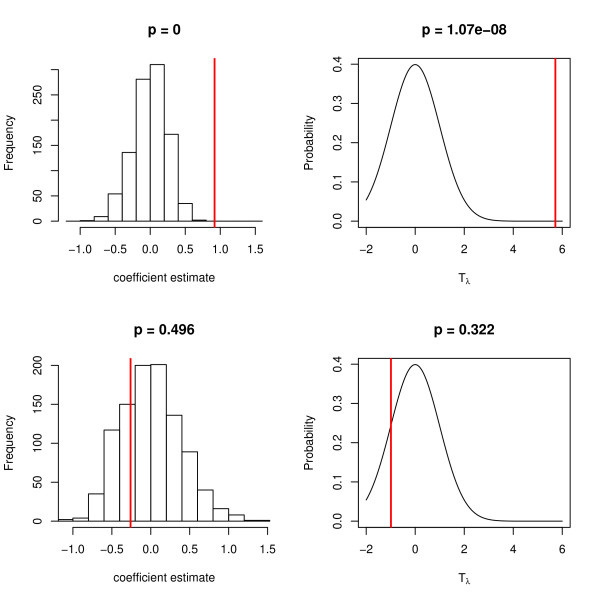
**Null distributions of the regression coefficients and tests statistics in the ridge regression model for data with continuous outcomes**. Left column - histogram of fitted coefficients obtained from the permutation test, with the coefficient fitted to the original data indicated by a vertical line. Right column - Null distribution of the test statistic used in the approximate test (a standard normal distribution), with the test statistic of the fitted coefficient indicated by a vertical line. Top row, a SNP associated with phenotype; bottom row, a SNP not associated with phenotype. The causal and non-causal SNPs are from the same replicate in the simulation study where *n *= 500, *m *= 20, *λ *= 1. *p*-values are shown above each plot.

Genotypes and corresponding phenotypes were generated as described above, for a range of data sets dimensions: *n *= 500, 1000, 5000 and *m *= 20, 100, 1000 and all SNPs in the 1 Mb region (approximately 10,000 SNPs). Phenotypes were generated as $Y_{i}\sim N\left(1+2k,1\right)$ with *k *being the minor allele count at the causal SNP. A range of values of the shrinkage parameter *λ *were used: *λ *= 0.1, 1, 10, 100. In Figure 3, the left column shows the null distributions, generated in a permutation test, used to estimate the significance of a ridge regression coefficient for a significant SNP (top row) and for a SNP that is not associated with phenotype (bottom row). The coefficient fitted to the original data is indicated. In the right column, the null distribution of the test statistic used in the approximate test is shown, with the test statistic of the fitted coefficient indicated. Ridge regression models were fitted using the shrinkage parameter *λ *= 1. These results are examples taken from a single simulation, and above each plot the *p*-value according to the permutation test (left) or the approximate test (right) is shown.

Table 2 compares the performance of the approximate and permutation tests in different simulation scenarios and at different values of the shrinkage parameter. We see that the approximate test performs well compared to the permutation test in terms of power (true positive rate) and that it has a slightly higher false positive rate.

**Table 2:** Performance comparison in simulated data with continuous outcomes

			Shrinkage Parameter							
			Approximate test				Permutation test			
			**0.1**	**1**	**10**	**100**	**0.1**	**1**	**10**	**100**
**Individuals**	**SNPs**									
500	20									
		TPR	1.000	1.000	1.000	1.000	1.000	1.000	1.000	1.000
		FPR	0.045	0.045	0.061	0.133	0.015	0.015	0.017	0.095
	100									
		TPR	1.000	1.000	1.000	1.000	1.000	1.000	1.000	1.000
		FPR	0.056	0.054	0.071	0.141	0.015	0.018	0.024	0.074
	1000									
		TPR	0.100	0.500	0.900	1.000	0.000	0.200	0.800	1.000
		FPR	0.038	0.045	0.049	0.080	0.007	0.006	0.010	0.029
	ALL									
		TPR	1.000	1.000	1.000	1.000	1.000	1.000	1.000	1.000
		FPR	0.318	0.071	0.068	0.069	0.019	0.019	0.020	0.020
1000	20									
		TPR	0.900	1.000	1.000	1.000	0.900	1.000	1.000	1.000
		FPR	0.043	0.043	0.087	0.137	0.013	0.013	0.034	0.096
	100									
		TPR	0.900	1.000	1.000	1.000	0.900	0.900	1.000	1.000
		FPR	0.051	0.052	0.060	0.108	0.023	0.023	0.019	0.062
	1000									
		TPR	0.700	0.700	1.000	1.000	0.400	0.500	0.900	1.000
		FPR	0.060	0.058	0.055	0.076	0.007	0.008	0.010	0.020
	ALL									
		TPR	1.000	1.000	1.000	1.000	1.000	1.000	1.000	1.000
		FPR	0.166	0.155	0.110	0.071	0.015	0.015	0.015	0.017
5000	20									
		TPR	1.000	1.000	1.000	1.000	1.000	1.000	1.000	1.000
		FPR	0.048	0.048	0.048	0.113	0.006	0.006	0.006	0.053
	100									
		TPR	0.900	0.900	1.000	1.000	0.800	0.900	1.000	1.000
		FPR	0.055	0.052	0.062	0.100	0.003	0.001	0.007	0.055
	1000									
		TPR	0.700	0.700	1.000	1.000	0.700	0.700	0.900	1.000
		FPR	0.046	0.046	0.045	0.060	0.006	0.007	0.008	0.014
	ALL									
		TPR	0.400	0.500	0.900	1.000	0.300	0.900	0.900	1.000
		FPR	0.026	0.027	0.029	0.042	0.007	0.007	0.007	0.009

Performance comparison between a permutation test and the approximate test. Data are simulated genotype data with continuous phenotypes. Reported are proportion of true positive and false positive results at significance threshold *α *= 0.05. **TPR **= True Positive Rate, **FPR **= False positive rate. Results for each simulation scenario are averaged over ten replicates.

When ranking the SNPs in order of significance, the approximate test and the permutation test ranked the SNPs identically or nearly so (results not shown).

**Figure 4:**
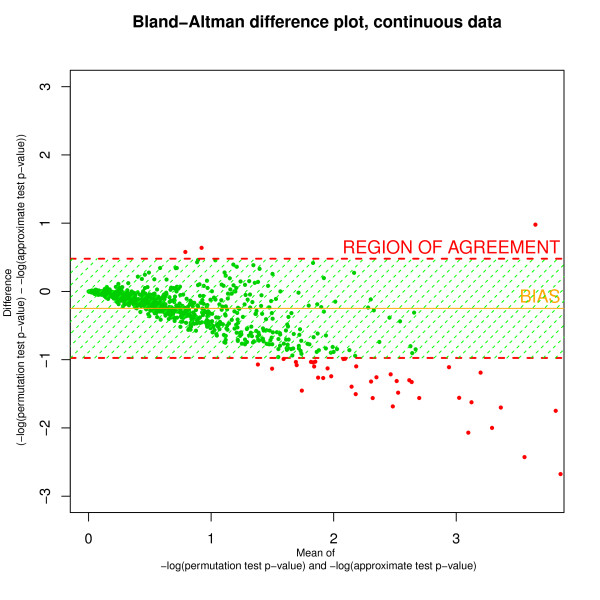
**Bland-Altman plot of mean versus difference for ***p***-values computed using both tests**. *p*-values were computed using both the approximate test and a the permutation test for data with continuous outcomes, and are plotted on the -log scale. *n *= 5000, *m *= 1000, *λ *= 0.1.

Figure 4 shows a Bland-Altman plot [28] of difference (permutation test *p*-value - *z*-type test *p*-value) against mean for the *p*-values of 1000 SNPs in 5000 individuals. *p*-values are plotted on the -log scale. We see that the bias is towards smaller *p*-values from the approximate test, which is congruous with the higher false positive rate for the approximate test shown in Table 2.

### Continuous Phenotypes with Multiple Causal SNPs

For complex diseases, multiple causal SNPs are likely to affect the phenotype. We investigated the performance of the test when more than one SNP is associated with phenotype. We simulated data from two different scenarios: *n *= 500, *m *= 100 and *n *= 500, *m *= 1000. In each region of *m *simulated genotypes, ten SNPs with minor allele frequency 0.10 *- *0.15 were designated causal and given effect size 1; the non-causal SNPs had effect size 0. Phenotypes were simulated as **Y **= **X*β ***+ ***ε***, $\varepsilon \sim N\left(0,\sigma^{2}I\right)$, *σ*^2 ^= 1 where ***β ***is the vector of effect sizes.

Results are presented in Table 3. We see that the conclusions drawn about the test, of adequate power at the cost of a slightly higher false positive rate, are equally valid when multiple SNPs in the data are associated with phenotype.

**Table 3:** Multiple causal phenotypes

		Approximate test				Permutation Test			
		**0.1**	**1**	**10**	**100**	**0.1**	**1**	**10**	**100**
**SNPs**									
**100**	TPR	0.624	0.717	0.939	1.000	0.252	0.312	0.517	0.910
	FPR	0.064	0.061	0.091	0.250	0.001	0.001	0.004	0.078
**1000**	TPR	0.210	0.250	0.670	0.970	0.020	0.070	0.170	0.770
	FPR	0.074	0.058	0.060	0.100	0.000	0.000	0.001	0.011

Performance comparison between a permutation test and the approximate test in simulated genotype data with continuous phenotypes and multiple causal SNPs. Reported are proportion of true positive and false positive results at significance threshold *α *= 0.05. *n *= 500 and in each scenario ten SNPs are with MAF 0.10 - 0.15 are designated causal with effect size 1; the rest of the SNPs have 0 effect size. **TPR **= True Positive Rate, **FPR **= False positive rate. Results for each simulation scenario are averaged over ten replicates.

### Computational performance comparison

Using an example simulation, we compared the computational time required to compute the approximate and the permutation tests. A data set with dimensions *n *= 1000, *m *= 1000 and *λ *= 1 was used. Approximate test and permutation test *p - *values were computed and the time taken to arrive at the *p*-values was recorded. Calculations were done using R version 2.12.0 [27] on an iMac running Mac OS X Version 10.6.7, fitted with an 2.8 Ghz Intel Core i7 processor and 16 GB 1067 MHz DDr3 RAM. Computational times are compared in Table 4. We see that the permutation test takes approximately 500 times longer to compute than the approximate test.

**Table 4:** Comparison of computational performance

	Approximate test	Permutation test
time (seconds)	1.936	1043.604

Comparison of computation performance of approximate test and permutation test. *n *= 1000, *m *= 1000 and *λ *= 1. Approximate test and permutation test *p - *values were computed and the time taken to arrive at the *p*-values was recorded. Calculations were done using R version 2.12.0 [27] on an iMac running Mac OS X Version 10.6.7, fitted with an 2.8 Ghz Intel Core i7 processor and 16 GB 1067 MHz DDr3 RAM.

### Binary phenotypes

Genotypes and corresponding binary phenotypes were generated for nine different data set dimensions: *n *= 500, 5000 and *m *= 20, 100, 1000, 2000, and *n *= 500, *m *= all SNPs in the 1 Mb region (approximately 10,000 SNPs). The genotype relative risk, *r*, was specified as 2. For the largest data dimensions (*n *= 500, *m *≈ 10, 000), results are not shown for the permutation test, due to the computational time required to fit ridge regression models on data of this size. Similarly, results are not shown when *n *= 5000 and *m *≈ 10, 000.

**Figure 5:**
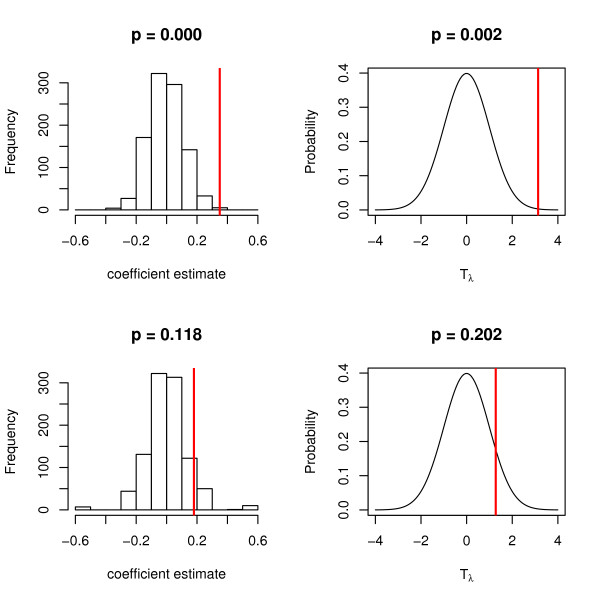
**Null distributions of the regression coefficients and tests statistics in the ridge regression model for data with binary outcomes**. Left column - histogram of fitted coefficients in the permutation test, with the coefficient fitted to the original data indicated by a vertical line. Right column - Null distribution of the test statistic used in the approximate test (a standard normal distribution) with the test statistic of the fitted coefficient indicated by a vertical line. Top row, a SNP associated with phenotype; bottom row, a SNP not associated with phenotype. The causal and non-causal SNPs are from the same replicate in the simulation study where *n *= 500, *m *= 20, *λ *= 1. *p*-values are shown above each plot.

In Figure 5, the left column shows the null distributions, generated in a permutation test, used to estimate the significance of a ridge regression coefficient for a significant SNP (top row) and for a SNP that is not associated with (bottom row) in data with binary outcomes. The coefficient fitted to the original data is indicated. In the right column, the null distribution of the test statistic used in the approximate test is shown, with the test statistic of the fitted coefficient indicated. Ridge regression models were fitted using the shrinkage parameter *λ *= 1. These results are examples taken from a single simulation, and above each plot the *p*-value according to the permutation test (left) or the approximate test (right) is shown.

**Figure 6:**
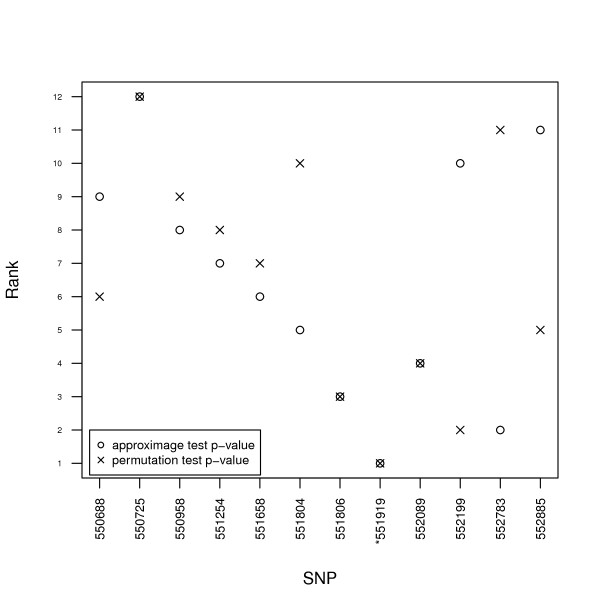
**Comparison of the ranking of SNPs**. SNPs are ranked by *p*-value, from 1 (most significant) to least significant, by both the approximate test and the permutation test. Data are genotypes with binary outcomes. The SNP name marked with an asterisk * is the causal SNP, and is the most significant SNP according to both tests. *n *= 500, *m *= 20, *λ *= 1. Only twelve SNPs are shown because the SNPs that were invariant among the sampled individuals were removed from the data before analysis.

Figure 6 compares the ranking of the SNPs from most significant (rank = 1) to least significant. Only twelve SNPs are shown because the SNPs that were invariant in the data were removed before analysis. The SNPs were ranked according to both the approximate test and the permutation test. From Figure 6 we see that whilst the ranking of the SNPs was not identical, the most strongly associated SNPs are ranked as such by both tests.

Table 5 compares the performance of the approximate test and a permutation test of significance for different sized data sets and at different values of the shrinkage parameter: *λ *= 0.1, 1, 10, 100. For most data set dimensions and values of *λ*, the *z*-type test is more conservative than the permutation test, with lower true positive and false positive rates. This is in contrast to the linear regression case.

**Table 5:** Performance comparison in simulated data with binary outcomes

			Shrinkage Parameter							
			Approximate test				Permutation test			
			**0.1**	**1**	**10**	**100**	**0.1**	**1**	**10**	**100**
**Individuals**	**SNPs**									
500	20									
		TPR	0.300	0.500	0.900	0.900	0.400	0.600	0.900	0.900
		FPR	0.023	0.036	0.068	0.142	0.078	0.078	0.099	0.174
	100									
		TPR	0.100	0.100	0.500	0.900	0.200	0.200	0.400	0.900
		FPR	0.024	0.037	0.046	0.087	0.050	0.052	0.058	0.115
	1000									
		TPR	0.200	0.300	0.500	0.700	0.100	0.100	0.400	0.700
		FPR	0.103	0.096	0.071	0.054	0.046	0.045	0.047	0.056
	2000									
		TPR	0.000	0.300	0.500	0.700	0.200	0.300	0.300	0.700
		FPR	0.008	0.056	0.081	0.063	0.052	0.049	0.048	0.055
	ALL									
		TPR	0.000	0.000	0.600	0.900	-	-	-	-
		FPR	0.000	0.000	0.014	0.068	-	-	-	-
5000	20									
		TPR	0.700	0.800	1.000	1.000	0.700	0.800	1.000	1.000
		FPR	0.024	0.024	0.030	0.096	0.090	0.083	0.089	0.154
	100									
		TPR	0.400	0.400	0.900	1.000	0.200	0.300	0.900	1.000
		FPR	0.027	0.028	0.041	0.078	0.071	0.067	0.078	0.110
	1000									
		TPR	0.200	0.300	0.600	1.000	0.100	0.200	0.600	1.000
		FPR	0.047	0.046	0.041	0.053	0.053	0.052	0.052	0.062
	2000									
		TPR	0.000	0.200	0.500	1.000	0.000	0.100	0.400	1.000
		FPR	0.074	0.067	0.056	0.057	0.053	0.052	0.053	0.058

Performance comparison between a permutation test and the approximate test in simulated genotype data with case-control phenotypes. Reported are proportion of true positive and false positive results at significance threshold *α *= 0.05. **TPR **= True Positive Rate, **FPR **= False positive rate. Results for each simulation scenario are averaged over ten replicates.

### Comparison with univariate tests of significance

The performance of tests of significance of ridge regression coefficients, in terms of true and false positive rates, was compared to the performance of univariate tests. Comparisons were made in each of the simulation settings: the null simulation, and the simulations with continuous and with binary outcomes. The results are shown in Additional File 1, tables S3, S4 and S5. As would be expected when using a penalised regression approach such as ridge regression, the performance of the corresponding significance test depends on the degree of shrinkage, which is controlled by the shrinkage parameter *λ*. The performance of the approximate test is comparable to that of a permutation test, with the advantage of a much-reduced computational burden. Further, ridge regression has the advantage over univariate tests of significance that it results in a much lower false positive rate. These advantages of ridge regression compared to univariate methods are further illustrated in the study of lung cancer data which follows.

### Lung cancer data

Genome-wide association studies have identified SNPs associated with lung cancer disease status. SNPs have been identified at chromosomal locations 15q25 [29,30], 5p15 [31] and 6p21 [32]. The associations at 15q25 and 5p15 have been replicated in white populations, but the association at 6p21 has not [33]. Not all studies successfully replicated the associations at 15q25 and 5p15 [34].

Here, we use ridge regression to re-evaluate a set of 35 SNPs for association with lung cancer disease status. Whilst these data are not as high-dimensional as those from a genome-wide study, they allow us to illustrate the features of using ridge regression for genetic data. We show that ridge regression is a useful technique when data are correlated, and illustrate that multivariate methods have advantages over univariate tests of significance.

Data consist of genotypes and non-genetic predictors from approximately 4000 individuals in the European Prospective Investigation into Cancer and Nutrition (EPIC, [35]). Missing genotypes were imputed using mean imputation. Gender, smoking status and age were included as unconstrained parameters in the model.

For the purpose of comparison, univariate (SNP-by-SNP) *p*-values were calculated. Univariate *p*-values were calculated by fitting a logistic regression model for each SNP independently, with gender, smoking status and age included in the model. A Wald test was then used to estimate the significance of the coefficient.

In this example, *m < n *and (unpenalised) multivariate regression does give rise to unique parameter estimates. Multivariate *p*-values (equivalent to *p*-values from the *z*-type test for ridge regression coefficients with a ridge penalty of zero) are also reported here.

In a data set of this size, it is computationally feasible to fit a ridge regression model with a range of values of *λ*. The ridge trace (Figure 1) is a plot of parameter estimates against *λ *[11]. The ridge trace typically suggests a range of values of *λ *rather than a single best value. In Figure 1, *λ *= 150 seems to be the point at which the ridge estimates stabilise, and Table 6 reports the corresponding approximate *p*-values.

**Table 6:** Lung cancer data

SNP (chromosome)	Univariate *p*-value	Multivariate *p*-value unpenalised	Approximate *p*-value *λ *= 150	Permutation *p*-value *λ *= 150
**rs8034191 **(15q25)	0.009	0.603	0.007	0.006

**rs16969968 **(15q25)	0.001	0.183	0.001	0.000

**rs402710 **(5p15)	0.213	0.012	0.011	0.004

**rs4324798 **(6p21)	0.513	0.231	0.248	0.251

Of the 35 SNPs in the lung cancer data set, four have been previously reported to be associated with lung cancer disease status. For these four SNPs, this table reports univariate, multivariate, approximate and permutation test *p*-values.

Figure 1 shows the ridge trace, and Figure 2 shows a plot of *p*-values with increasing *λ*. Due to low LD between most of the SNPs, most coefficient estimates do not change significantly with increasing *λ *and nor do estimates of their significance. SNPs rs8034191 and rs16969968 are both located at 15q25 and are in high LD (r^2 ^= 0.961 in HapMap CEU population, r^2 ^= 0.81 in our data). In contrast to most of the SNPs, coefficient estimates for these two SNPs do change rapidly with change in the shrinkage parameter.

Further, with increasing shrinkage and stabilisation of the estimates, the approximate *p*-values for these SNPs become significant (Figure 2). rs402710, which is not in LD with other SNPs in the data, is significant in a multiple logistic regression model even when no penalty is included in the model (*λ *= 0). A further SNP, rs671330, in chromosome 6, has a nominally significant *p*-value in the approximate test (ranging from 0.048 to 0.040 with increased shrinkage), but again this SNP has not previously been shown to be associated with lung cancer risk.

The *p*-value trace using permutation test *p*-values (Figure 2b) shows good agreement with the approximate *p*-value trace (Figure 2a). Figure 2b is much more computationally expensive to produce than Figure 2a, thus the use of the approximate test makes the plotting of a *p*-value trace for a range of values of *λ *more feasible.

Table 6 presents the univariate, multivariate, approximate and permutation test *p*-values at *λ *= 150 for four SNPs from the regions which have previously been shown to be associated with lung cancer disease status. Using ridge regression, we replicate the previously found associations at 15q25 and 5p15, but fail to replicate the association at 6p21. Using the univariate test, a further SNP, rs6746834 (on chromosome 2) was nominally significant at the 0.05 level (*p *= 0.049), but association at this region has not previously been shown. This SNP was not found to be significantly associated with lung cancer disease status by the approximate test. We interpret this as a false positive that arises when univariate tests are used.

The results in Table 6 demonstrate the advantage of multivariate tests, and specifically of ridge regression, over univariate tests of significance. SNP rs402710, which has previously been shown to be associated with lung cancer disease status [31], was not found to be significant using the univariate test but was found as such by the multivariate methods. The two SNPs that are correlated, rs8034191 and rs16969968, were not significant in multiple regression but were significant in ridge regression, demonstrating the advantage of using ridge regression when SNPs are correlated.

These results demonstrate that this approximate test of significance for coefficients fitted using logistic ridge regression reproduces previously ascertained associations, at reduced computational cost compared to a permutation test, even when SNPs are highly correlated.

## Conclusions

We present and evaluate the performance of a test of significance for coefficients estimated using ridge regression. We evaluate the test as applied to both linear and logistic ridge regression models. Our evaluation is by means of simulation studies across a range of scenarios representative of genetic data. We evaluate the test by comparing its performance to that of a permutation test.

We evaluate the performance of the test when it is applied to a real data set. The data set comprises lifestyle data and genotypes together with lung cancer case-control status. Using the proposed test, we successfully replicate previously found associations at much reduced computational cost compared to a permutation test. This demonstrates the utility of the test for detecting significant variables when predictor variables are highly correlated, as were two significant SNPs in the lung cancer data.

Stability selection [36] is a method for variable selection that has received attention in the literature in recent years. It combines subsampling of the data with a dimension reduction technique, with the aim of finding consistently significant variables. Penalised regression methods that perform variable selection, such as the Lasso [10] and the Elastic Net [37], have been used in stability selection. To use ridge regression with stability selection, a way of determining which variables to select in each subsample of the data is required. A permutation test would be computationally expensive in this context, rapidly becoming infeasible if for large *n*, if the guidelines of 100 subsamples of size *n*/2 given by Meinshausen & Bühlmann [36] were followed. The test of significance proposed here, being much less computationally expensive than a permutation test, makes the combination of ridge regression and stability selection a feasible possibility.

The choice of shrinkage parameter in ridge regression is discussed in the literature, but no consensus method provides an universally optimum choice. The proposed test performs well over a range of values of *λ*. We introduce the *p*-value trace, a plot of the change in the negative logarithm of the *p*-value of the regression coefficients as the shrinkage parameter increases from zero. This trace can be used in combination with the ridge trace of [11] to visualise how the significance of the regression parameters, as well as their value, changes with increasing shrinkage. Such a plot could aid the identification of significant coefficients in the ridge model.

## Authors' contributions

EC ran the simulations, analysed the data, and wrote the paper. PV was involved in the collection of the lung cancer data, supervised the analysis, and read and commented on the draft. MDI came up with the idea, supervised the work, and commented on drafts of the paper. All authors read and approved the final manuscript.

## Supplementary Material

Additional file 1 **Table S1 **- **Performance comparison in null simulation using t-type test**. **Table S2 **- Performance comparison in simulated data with continuous outcomes using t-type test. **Table S3 **- Performance comparison in null simulation including comparison to univariate tests of significance. **Table S4 **- Performance comparison with continuous outcomes including comparison to univariate tests of significance. **Table S5 **- Performance comparison with binary outcomes including comparison to univariate tests of significance. Click here for file
