# Supplementary material for: Significance testing in ridge regression for genetic data
Source: BMC Bioinformatics. 2011 Sep 19;12:372. doi: 10.1186/1471-2105-12-372 (PMC3228544; doi:10.1186/1471-2105-12-372)
Supplement: Additional file 1 — Table S1 - Performance comparison in null simulation using t-type test. Table S2 - Performance comparison in simulated data with continuous outcomes using t-type test. Table S3 - Performance comparison in null simulation including comparison to univariate tests of significance. Table S4 - Performance comparison with continuous outcomes including comparison to univariate tests of significance. Table S5 - Performance comparison with binary outcomes including comparison to univariate tests of significance. [file 1471-2105-12-372-S1.PDF]

**Table S1 - Performance comparison in null simulation using t-type test**

| | | Shrinkage Paramter | | | | | | | |
|---|---|---|---|---|---|---|---|---|---|
| | | **Normal test** | | | | ***t*-test** | | | |
| | | 0.1 | 1 | 10 | 100 | 0.1 | 1 | 10 | 100 |
| **Individuals** | **SNPs** | | | | | | | | |
| 500 | 20 | 0.066 | 0.066 | 0.066 | 0.075 | 0.066 | 0.066 | 0.066 | 0.066 |
| 1000 | 1000 | 0.050 | 0.049 | 0.050 | 0.046 | 0.049 | 0.048 | 0.049 | 0.046 |

False positive rates at the nominal significance threshold $\alpha = 0.05$ in null datasets, comparing the performance of the approximate test when the normal or $t$-distribution is used as the null distribution of the test statistic.

**Table S2 - Performance comparison in simulated data with continuous outcomes using t-type test**

| | | | Shrinkage Paramter | | | | | | | |
|---|---|---|---|---|---|---|---|---|---|---|
| | | | **Normal test** | | | | ***t*-test** | | | |
| | | | 0.1 | 1 | 10 | 100 | 0.1 | 1 | 10 | 100 |
| **Individuals** | **SNPs** | | | | | | | | | |
| 500 | 20 | | | | | | | | | |
| | | TPR | 1.000 | 1.000 | 1.000 | 1.000 | 1.000 | 1.000 | 1.000 | 1.000 |
| | | FPR | 0.045 | 0.045 | 0.061 | 0.133 | 0.038 | 0.038 | 0.047 | 0.118 |
| | 100 | | | | | | | | | |
| | | TPR | 1.000 | 1.000 | 1.000 | 1.000 | 1.000 | 1.000 | 1.000 | 1.000 |
| | | FPR | 0.056 | 0.054 | 0.071 | 0.141 | 0.046 | 0.044 | 0.063 | 0.134 |
| 1000 | 20 | | | | | | | | | |
| | | TPR | 0.900 | 1.000 | 1.000 | 1.000 | 0.900 | 1.000 | 1.000 | 1.000 |
| | | FPR | 0.043 | 0.043 | 0.087 | 0.137 | 0.034 | 0.034 | 0.071 | 0.119 |
| | 100 | | | | | | | | | |
| | | TPR | 0.900 | 1.000 | 1.000 | 1.000 | 0.900 | 1.000 | 1.000 | 1.000 |
| | | FPR | 0.051 | 0.052 | 0.060 | 0.108 | 0.048 | 0.050 | 0.056 | 0.099 |
| | 1000 | | | | | | | | | |
| | | TPR | 0.700 | 0.700 | 1.000 | 1.000 | 0.700 | 0.700 | 1.000 | 1.000 |
| | | FPR | 0.060 | 0.058 | 0.055 | 0.076 | 0.059 | 0.058 | 0.055 | 0.075 |
| 5000 | 20 | | | | | | | | | |
| | | TPR | 1.000 | 1.000 | 1.000 | 1.000 | 1.000 | 1.000 | 1.000 | 1.000 |
| | | FPR | 0.048 | 0.048 | 0.048 | 0.113 | 0.036 | 0.036 | 0.031 | 0.096 |
| | 100 | | | | | | | | | |
| | | TPR | 0.900 | 0.900 | 1.000 | 1.000 | 0.900 | 0.900 | 1.000 | 1.000 |
| | | FPR | 0.055 | 0.052 | 0.062 | 0.100 | 0.052 | 0.047 | 0.059 | 0.096 |
| | 1000 | | | | | | | | | |
| | | TPR | 0.700 | 0.700 | 1.000 | 1.000 | 0.700 | 0.700 | 1.000 | 1.000 |
| | | FPR | 0.046 | 0.046 | 0.045 | 0.060 | 0.046 | 0.045 | 0.045 | 0.060 |

Performance comparison of the approximate test using a normal or $t$-distribution as the null distribution of

the test statistic in simulated genotype data with continuous phenotypes. Reported are proportion of true positive and false positive results at significance threshold $\alpha = 0.05$. **TPR**=True Positive Rate, **FPR**=False positive rate. Results for each simulation scenario are averaged over ten replicates.

**Table S3 - Performance comparison in null simulation including comparison to univariate tests of significance**

| | | | | **Shrinkage Paramter** | | | | | | | |
|---|---|---|---|---|---|---|---|---|---|---|---|
| | | | **Univariate test** | **Approximate test** | | | | **Permutation test** | | | |
| | | | | 0.1 | 1 | 10 | 100 | 0.1 | 1 | 10 | 100 |
| **Individuals** | **SNPs** | **Outcomes** | | | | | | | | | |
| 500 | 20 | Continuous | 0.106 | 0.066 | 0.066 | 0.066 | 0.075 | 0.066 | 0.066 | 0.066 | 0.075 |
| | | Binary | 0.052 | 0.021 | 0.021 | 0.041 | 0.067 | 0.027 | 0.027 | 0.033 | 0.052 |
| 1000 | 1000 | Continuous | 0.050 | 0.050 | 0.049 | 0.050 | 0.046 | 0.051 | 0.052 | 0.050 | 0.046 |
| | | Binary | 0.026 | 0.118 | 0.092 | 0.066 | 0.053 | 0.054 | 0.056 | 0.053 | 0.051 |

Performance comparison in null simulation including comparison to univariate tests of significance. False positive rates at the nominal significance threshold $\alpha = 0.05$ in null datasets. In each scenario, results are averaged over ten replicates.

**Table S4 - Performance comparison with continuous outcomes including comparison to univariate tests of significance**

| | | | | Shrinkage Paramter | | | | | | | |
|---|---|---|---|---|---|---|---|---|---|---|---|
| | | | **Univariate** | **Approximate test** | | | | **Permutation test** | | | |
| | | | **test** | 0.1 | 1 | 10 | 100 | 0.1 | 1 | 10 | 100 |
| **Individuals** | **SNPs** | | | | | | | | | | |
| 500 | 20 | | | | | | | | | | |
| | | TPR | 1.000 | 1.000 | 1.000 | 1.000 | 1.000 | 1.000 | 1.000 | 1.000 | 1.000 |
| | | FPR | 0.341 | 0.045 | 0.045 | 0.061 | 0.133 | 0.015 | 0.015 | 0.017 | 0.095 |
| | 100 | | | | | | | | | | |
| | | TPR | 1.000 | 1.000 | 1.000 | 1.000 | 1.000 | 1.000 | 1.000 | 1.000 | 1.000 |
| | | FPR | 0.331 | 0.056 | 0.054 | 0.071 | 0.141 | 0.015 | 0.018 | 0.024 | 0.074 |
| | 1000 | | | | | | | | | | |
| | | TPR | 1.000 | 0.100 | 0.500 | 0.900 | 1.000 | 0.000 | 0.200 | 0.800 | 1.000 |
| | | FPR | 0.219 | 0.038 | 0.045 | 0.049 | 0.080 | 0.007 | 0.006 | 0.010 | 0.029 |
| | ALL | | | | | | | | | | |
| | | TPR | 1.000 | 1.000 | 1.000 | 1.000 | 1.000 | 1.000 | 1.000 | 1.000 | 1.000 |
| | | FPR | 0.100 | 0.318 | 0.071 | 0.068 | 0.069 | 0.019 | 0.019 | 0.020 | 0.020 |
| 1000 | 20 | | | | | | | | | | |
| | | TPR | 1.000 | 0.900 | 1.000 | 1.000 | 1.000 | 0.900 | 1.000 | 1.000 | 1.000 |
| | | FPR | 0.348 | 0.043 | 0.043 | 0.087 | 0.137 | 0.013 | 0.013 | 0.034 | 0.096 |
| | 100 | | | | | | | | | | |
| | | TPR | 1.000 | 0.900 | 1.000 | 1.000 | 1.000 | 0.900 | 0.900 | 1.000 | 1.000 |
| | | FPR | 0.432 | 0.051 | 0.052 | 0.060 | 0.108 | 0.023 | 0.023 | 0.019 | 0.062 |
| | 1000 | | | | | | | | | | |
| | | TPR | 1.000 | 0.700 | 0.700 | 1.000 | 1.000 | 0.400 | 0.500 | 0.900 | 1.000 |
| | | FPR | 0.295 | 0.060 | 0.058 | 0.055 | 0.076 | 0.007 | 0.008 | 0.010 | 0.020 |
| | ALL | | | | | | | | | | |
| | | TPR | 1.000 | 1.000 | 1.000 | 1.000 | 1.000 | 1.000 | 1.000 | 1.000 | 1.000 |
| | | FPR | 0.114 | 0.166 | 0.155 | 0.110 | 0.071 | 0.015 | 0.015 | 0.015 | 0.017 |
| 5000 | 20 | | | | | | | | | | |
| | | TPR | 1.000 | 1.000 | 1.000 | 1.000 | 1.000 | 1.000 | 1.000 | 1.000 | 1.000 |
| | | FPR | 0.541 | 0.048 | 0.048 | 0.048 | 0.113 | 0.006 | 0.006 | 0.006 | 0.053 |
| | 100 | | | | | | | | | | |
| | | TPR | 1.000 | 0.900 | 0.900 | 1.000 | 1.000 | 0.800 | 0.900 | 1.000 | 1.000 |
| | | FPR | 0.498 | 0.055 | 0.052 | 0.062 | 0.100 | 0.003 | 0.001 | 0.007 | 0.055 |
| | 1000 | | | | | | | | | | |
| | | TPR | 1.000 | 0.700 | 0.700 | 1.000 | 1.000 | 0.700 | 0.700 | 0.900 | 1.000 |
| | | FPR | 0.397 | 0.046 | 0.046 | 0.045 | 0.060 | 0.006 | 0.007 | 0.008 | 0.014 |
| | ALL | | | | | | | | | | |
| | | TPR | 1.000 | 0.400 | 0.500 | 0.900 | 1.000 | 0.300 | 0.900 | 0.900 | 1.000 |
| | | FPR | 0.216 | 0.026 | 0.027 | 0.029 | 0.042 | 0.007 | 0.007 | 0.007 | 0.009 |

Performance comparison between a univariate test, a permutation test and the approximate test in simulated genotype data with continuous phenotypes. Reported are proportion of true positive and false positive results at significance threshold $\alpha = 0.05$. **TPR**=True Positive Rate, **FPR**=False positive rate. Results for each simulation scenario are averaged over ten replicates.

**Table S5 - Performance comparison with binary outcomes including comparison to univariate tests of significance**

| | | | | Shrinkage Paramter | | | | | | | |
|---|---|---|---|---|---|---|---|---|---|---|---|
| | | | **Univariate test** | **Approximate test** | | | | **Permutation test** | | | |
| | | | | 0.1 | 1 | 10 | 100 | 0.1 | 1 | 10 | 100 |
| **Individuals** | **SNPs** | | | | | | | | | | |
| 500 | 20 | | | | | | | | | | |
| | | TPR | 1.000 | 0.300 | 0.500 | 0.900 | 0.900 | 0.400 | 0.600 | 0.900 | 0.900 |
| | | FPR | 0.134 | 0.023 | 0.036 | 0.068 | 0.142 | 0.078 | 0.078 | 0.099 | 0.174 |
| | 100 | | | | | | | | | | |
| | | TPR | 1.0000 | 0.100 | 0.100 | 0.500 | 0.900 | 0.200 | 0.200 | 0.400 | 0.900 |
| | | FPR | 0.134 | 0.024 | 0.037 | 0.046 | 0.087 | 0.050 | 0.052 | 0.058 | 0.115 |
| | 1000 | | | | | | | | | | |
| | | TPR | 1.000 | 0.200 | 0.300 | 0.500 | 0.700 | 0.100 | 0.100 | 0.400 | 0.700 |
| | | FPR | 0.063 | 0.103 | 0.096 | 0.071 | 0.054 | 0.046 | 0.045 | 0.047 | 0.056 |
| | 2000 | | | | | | | | | | |
| | | TPR | 1.000 | 0.000 | 0.300 | 0.500 | 0.700 | 0.200 | 0.300 | 0.300 | 0.700 |
| | | FPR | 0.052 | 0.008 | 0.056 | 0.081 | 0.063 | 0.052 | 0.049 | 0.048 | 0.055 |
| | ALL | | | | | | | | | | |
| | | TPR | 1.000 | 0.000 | 0.000 | 0.600 | 0.900 | – | – | – | – |
| | | FPR | 0.033 | 0.000 | 0.000 | 0.014 | 0.068 | – | – | – | – |
| 5000 | 20 | | | | | | | | | | |
| | | TPR | 1.000 | 0.700 | 0.800 | 1.000 | 1.000 | 0.700 | 0.800 | 1.000 | 1.000 |
| | | FPR | 0.294 | 0.024 | 0.024 | 0.030 | 0.096 | 0.090 | 0.083 | 0.089 | 0.154 |
| | 100 | | | | | | | | | | |
| | | TPR | 1.000 | 0.400 | 0.400 | 0.900 | 1.000 | 0.200 | 0.300 | 0.900 | 1.000 |
| | | FPR | 0.271 | 0.027 | 0.028 | 0.041 | 0.078 | 0.071 | 0.067 | 0.078 | 0.110 |
| | 1000 | | | | | | | | | | |
| | | TPR | 1.000 | 0.200 | 0.300 | 0.600 | 1.000 | 0.100 | 0.200 | 0.600 | 1.000 |
| | | FPR | 0.176 | 0.047 | 0.046 | 0.041 | 0.053 | 0.053 | 0.052 | 0.052 | 0.062 |
| | 2000 | | | | | | | | | | |
| | | TPR | 1.000 | 0.000 | 0.200 | 0.500 | 1.000 | 0.000 | 0.100 | 0.400 | 1.000 |
| | | FPR | 0.136 | 0.074 | 0.067 | 0.056 | 0.057 | 0.053 | 0.052 | 0.053 | 0.058 |

Performance comparison between a univariate test, a permutation test and the approximate test in simulated genotype data with continuous phenotypes. Reported are proportion of true positive and false positive results at significance threshold $\alpha = 0.05$. **TPR**=True Positive Rate, **FPR**=False positive rate. Results for each simulation scenario are averaged over ten replicates.
